# Mediterranean Diet: A Tool to Break the Relationship of Atrial Fibrillation with the Metabolic Syndrome and Non-Alcoholic Fatty Liver Disease

**DOI:** 10.3390/nu14061260

**Published:** 2022-03-16

**Authors:** Flavio Giuseppe Biccirè, Tommaso Bucci, Danilo Menichelli, Vittoria Cammisotto, Pasquale Pignatelli, Roberto Carnevale, Daniele Pastori

**Affiliations:** 1 Department of General and Specialized Surgery “Paride Stefanini”, Sapienza University of Rome, 00161 Rome, Italy; flaviogiuseppe.biccire@uniroma1.it (F.G.B.); tommaso.bucci@uniroma1.it (T.B.); 2 Department of Clinical Internal, Anesthesiological and Cardiovascular Sciences, Sapienza University of Rome, 00161 Rome, Italy; danilo.menichelli@uniroma1.it (D.M.); vittoria.cammisotto@uniroma1.it (V.C.); pasquale.pignatelli@uniroma1.it (P.P.); 3 Mediterranea Cardiocentro, 80122 Naples, Italy; roberto.carnevale@uniroma1.it; 4 Department of Medical-Surgical Sciences and Biotechnologies, Sapienza University of Rome, 04100 Latina, Italy

**Keywords:** diet, atrial fibrillation, metabolic syndrome, NAFLD

## Abstract

Atrial fibrillation (AF) is the most common supraventricular arrhythmia associated with increased cardiovascular and non-cardiovascular morbidity and mortality. As multiple factors may predispose the onset of AF, the prevention of the occurrence, recurrence and complications of this arrhythmia is still challenging. In particular, a high prevalence of cardio-metabolic comorbidities such as the metabolic syndrome (MetS) and in its hepatic manifestation, the non-alcoholic fatty liver disease (NAFLD), have been described in the AF population. A common pathogenetic mechanism linking AF, MetS and NAFLD is represented by oxidative stress. For this reason, in the past decades, numerous studies have investigated the effect of different foods/nutrients with antioxidant properties for the prevention of, and their therapeutic role is still unclear. In this narrative comprehensive review, we will summarize current evidence on (1) the association between AF, MetS and NAFLD (2) the antioxidant role of Mediterranean Diet and its components for the prevention of AF and (3) the effects of Mediterranean Diet on MetS components and NAFLD.

## 1. Introduction

Atrial fibrillation (AF) represents the most frequently sustained arrhythmia and increases the individual risk of cardiovascular and non-cardiovascular death [1]. Thus, primary prevention of AF is of utmost importance for physicians and health systems. Nevertheless, AF recognizes a complex pathogenesis with the interaction of multiple risk factors, making population-based interventions to prevent this common arrhythmia challenging. Indeed, beside the increased risk of cardioembolic ischemic stroke, patients with AF have a twofold increased risk for premature death, myocardial infarction and heart failure compared to the general population [2,3,4]. The risk of cardiovascular events (CVEs) in AF still remains high when patients are anticoagulated with well-managed vitamin K antagonist or an appropriate direct oral anticoagulant [5,6,7].

This residual cardiovascular risk is conferred by the coexistence of multiple cardiovascular and metabolic risk factors that are frequently detected in patients with AF, such as the components of the metabolic syndrome (MetS) and non-alcoholic fatty liver disease (NAFLD). This implies that to reduce the cardiovascular risk, the therapeutic approach to patients with AF should not be limited to anticoagulation treatment but should also take into account the reach of therapeutic targets for concomitant comorbidities [8]. This concept has been recently underlined by the European Society of Cardiology guidelines which recommended an integrated approach consisting of anticoagulation, symptoms control, and proactive management of comorbidities according to the Atrial fibrillation Better Care pathway [1].

In this view, physical activity and adherence to a healthy dietary pattern represent the two cornerstones of non-pharmaceutical treatments. In the past years, several studies have investigated the possible relationship between dietary regimens and cardiac arrhythmias, especially AF [9]. Recent guidelines also further encourage the consumption of healthy dietary components, such as those of Mediterranean diet (Med-Diet), by means of their well-established effect in prolonging life expectancy and reducing cardiovascular burden [10]. However, the association between adherence to healthy eating habits and risk of AF is still unclear.

In this narrative review, we summarize current evidence on (1) the association between AF, MetS and NAFLD (2) the antioxidant role of Med-Diet and its components for the prevention of AF and (3) the effects of Med-Diet on MetS components and NAFLD.

## 2. Atrial Fibrillation, MetS, and NAFLD

There is a close relationship between MetS and AF; almost all components of MetS, including overweight, obesity, hypertension, dyslipidemia and diabetes have been associated with an increased risk of new-onset AF both in Asian and Western countries [11,12].

The MetS-associated dyslipidemia is characterized by low high-density lipoprotein (HDL) and high triglycerides, defining the so-called “atherogenic dyslipidemia”. In this context, it is noteworthy that blood levels of HDL and triglycerides, but not low-density lipoprotein (LDL), have been associated with an increased risk of AF [13,14].

MetS is highly prevalent in patients with AF, and its prevalence had a widely variability ranging between 21.8% in Eastern and 71.0% in Western countries (Table 1). In addition, the presence of MetS in AF patients increases the risk of CVEs [15]. Indeed, the MetS has been also included in a recent score, the 2MACE score (2 points for metabolic syndrome and age ≥ 75, and 1 point for myocardial infarction (MI) or revascularization, congestive heart failure (ejection fraction ≤ 40%) and thromboembolism (stroke or transient ischemic attack)) to predict cardiovascular complications in the AF population [16].

In addition to MetS, AF also shares some common risk factors with NAFLD, including oxidative stress and insulin resistance [17].

NAFLD defines a wide spectrum of disorders characterized by the deposition of fatty acids in the liver, causing inflammation and potentially leading to liver fibrosis and cirrhosis. The prevalence of NAFLD is high in Western Countries and it is estimated that about one third of the general population may be affected by this disorder [18].

There is a longstanding debate on whether NAFLD is associated with cardiovascular risk in virtue of its associated comorbidities or if it may represent an independent risk factor per se [19].

However, a growing body of evidence support the notion that NAFLD may elicit structural, electrical, and autonomic remodelling in the heart, representing a pro-arrhythmogenic substrate in the heart [20].

The reported prevalence of NAFLD in AF ranges from 27.4% to 42.2% in the largest cohort studies [21,22], as shown in Table 1. Of note, if AF and diabetes mellitus coexist, the prevalence of MetS rises to 88.2% (Table 1) [23]. Ultrasonographic criteria is most widely used in the studies, however, fatty liver index (FLI) may represent a valid alternative for the screening of NAFLD in large populations.

In addition, the prevalence of liver fibrosis, which represents the advanced stage of liver disease is between 5.5% and 6.8% in the AF population [24,25] (Table 1).

**Table 1:** Prevalence of liver fibrosis, non-alcoholic fatty liver disease (NAFLD) and metabolic syndrome in patient with atrial fibrillation.

	Study Design	Total Cohort	Prevalence (%)	Diagnostic Criteria
NAFLD				
Long [21]	CS	62	27.4	Computed tomography
Pastori [22]	P	1735	42.2	Fatty liver index (FLI) score ≥ 60
Zhang [26]	CS	39	46.2	Ultrasonography
Karajamaki [27]	P	36	50.0	Ultrasonography
Targher [23]	R	85	88.2	Ultrasonography
Metabolic syndrome				
Umetani [28]	P	592	21.8	ATP-III criteria
Xia [29]	P	137	32.1	The Chinese Medical Association Diabetes Branch (CMADB) and the National Cholesterol Education Program Third Adult Treatment Panel (NCEP-ATPIII) criteria
Vural [30]	R	161	46.0	Metabolic syndrome Working Group of The Society of Endocrinology and Metabolism of Turkey
Pastori [15]	P	1735	49.0	Modified ATP-III criteria
Mohanty [31]	P	1496	67.6	Diabetes plus any 2 other risk factors is sufficient for the diagnosis according to the World Health Organization
Ionin [32]	P	248	70.9	International Diabetes Federation (IDF) criteria
Decker [33]	P	1172	71.0	ATP III criteria
Liver fibrosis/Cirrhosis				
Karajamaki [27]	CC	36	-	Ultrasound elastography
Kang [24]	CS	59	6.8	NAFLD fibrosis score (NFS) and Fibrosis-4 (Fib-4) Index
Pastori [25]	P	2330	5.5	Fibrosis-4 (Fib-4) Index
Kuo [34]	R	289.559	3.6	International Classification of Diseases, 9th Revision (ICD-9)

CC: case–control; CS: cross sectional; P: prospective; R: retrospective; -: not applicable.

## 3. Oxidative Stress: A Common Milieu for AF and NAFLD

The high prevalence of cardio-metabolic risk factors detected in patients with AF and NAFLD contributes to the increased oxidative stress detected in these patients [35], that may represent a common pathogenetic mechanism linking AF and NAFLD.

Indeed, several studies have shown that patients with AF have an unbalanced oxidative status, characterized by high levels of reactive oxygen species (ROS) and unpaired enzymatic antioxidant activity, which may contribute to the high thrombotic risk [36,37]. ROS may cause damage to DNA, proteins, and lipids, induce tissue damage causing cardiac structural and electrical remodelling [38]. Moreover, several studies showed that an increase in oxidative stress is implicated not only in promoting AF but also in maintaining atrial arrhythmia [39]. Because ROS are characterized by a short half-life, the use of stable circulating markers, that reflect cellular and systemic oxidative stress, are usually assessed. Among these, are markers of lipid peroxidation (isoprostanes), oxidized phospholipids, malondialdehyde, nitrotyrosine, myeloperoxidase, and aminothiol compounds [40,41].

The increase in the ROS concentration and therefore of oxidative stress in AF may result from either the up-regulation of pro-oxidant enzymes, such as Nicotinamide Adenine Dinucleotide Phosphate Hydrogen (NADPH) () oxidase, or the down-regulation of antioxidant defences including glutathione peroxidase 3 (GPx3) and superoxide dismutase (SOD), which are among the most important circulating antioxidant enzymes [42].

A large prospective cohort study showed that the redox potentials of glutathione (E_h_GSH) and cysteine, markers of oxidative stress, were associated with the prevalence and incidence of AF. Particularly, the prevalence of AF was 30% higher for each 10% increase in E_h_GSH, while the same alteration resulted in a 40% increase in the risk of incident AF [43].

In a large cohort of AF patients, lower values of GPx3, the blood isoform of GPx, and SOD were detected in patients with CVEs, compared to those without. Moreover, a lower survival rate was observed in patients with GPx3 and SOD activities below the median than those above [44].

Regarding pro-oxidant pathways, in studies performed in animal models, superoxide and H_2_O_2_ produced from activated NADPH oxidase 2 (Nox2) and Nox4 isoforms were found to lead to myocyte apoptosis, fibrosis, and inflammation, which further promote AF perpetuation [45]. Furthermore, Nox2, the catalytic subunit of NADPH oxidase, is suggested to play a role in favoring the occurrence of AF via the formation of ROS. Serum sNox2-dp levels, a marker of Nox2 activation, were found significantly higher in patients with paroxysmal/persistent AF than in those with permanent AF [46]. Serum sNox2-dp levels were also measured in a prospective study including 1002 AF patients. The results showed that high Nox2 levels are predictive of CVEs, such as ischaemic stroke, myocardial infarction (MI), cardiac revascularization and transient ischaemic attack (TIA), and total mortality in patient with AF [47].

Oxidative stress also plays a pivotal role in the pathogenesis and progression of NAFLD. Indeed, oxidative stress may be the first hit of hepatic damage, according to “the two-hit hypothesis” [48], and ROS are the key mediators of hepatic damage and also in the progression of NAFLD to non-alcoholic steatohepatitis (NASH), thereby increasing lipid peroxidation, leading to tissue damage, inflammation and fibrogenesis [49,50]. A previous study showed that NAFLD patients had a higher serum level of Nox2 and isoprostanes as compared to patients with cardiovascular risk factors but without NAFLD [51]. Regarding antioxidant markers, reduced levels of the antioxidant vitamin E have been reported in NAFLD patients [52], and a cross-sectional study including 71 NAFLD and 171 non-NAFLD patients showed that plasma SOD was inversely associated with NAFLD [53].

## 4. Mediterranean Diet for the Prevention of AF

Considering the common comorbidities and pathogenetic mechanisms shared by AF, MetS and NAFLD, Med-Diet may have a beneficial effect on these conditions via both a direct antioxidant and a metabolic effect.

Indeed, the Med-Diet is characterized by a high intake of fruits, vegetables, legumes, monounsaturated fatty acids, including olive oil, and a moderate intake of fish and wine; it is very rich in antioxidant vitamins (β-carotene, vitamin C, vitamin E), natural folate, phytochemicals (flavonoids), and minerals such as selenium. The exact mechanism by which an increased adherence to the Med-Diet exerts its favourable effects is not known. The majority of data investigated the effect of single antioxidant foods/nutrients on the risk of AF.

### Antioxidant Foods/Nutrients Intake and Risk of AF

In a retrospective study on 800 subjects, Med-Diet adherence was found to be higher in patients without AF and, among AF patients, was associated with a more frequent spontaneous cardioversion of AF [54]. However, most of the evidence in the literature refers to single food consumption rather than dietary patterns. In Table 2, we report the largest prospective studies and randomized controlled trials (RCTs), thereby unravelling the “causal” or “casual” link between foods /nutrients and incident AF in patients free from AF at baseline.

One of the most investigated dietary factors for preventing AF are fish and long-chain omega-3 polyunsaturated fatty acids (PUFAs), which are well-represented elements of the Med-Diet. Previous experimental studies reported a protective effect of dietary fish oil against AF susceptibility in myocardiocytes of rabbits [55]. Accordingly, circulating levels of long-chain n-3 PUFAs and docosahexaenoic acid have been associated with a lower risk of incident AF [56].

Despite this, clinical studies investigating the role of fish/PUFAs dietary food intake in preventing AF have provided controversial data (Table 2).

Three metanalyses investigated these aspects and reported no major effects of fish or n-3 fatty acids on AF risk in both primary and secondary AF prevention [57,58,59]. Khawaja et al., included seven cohort studies and 11 RCTs investigating the incidence of AF in subjects with or without fish/fish oil or long-chain n-3 PUFAs consumption; the pooled odds ratio (OR) was 0.79 (95% confidence interval CI = 0.56–1.12; *p* = 0.19) for RCTs and 0.83 (95%CI 0.59–1.16; *p* = 0.27) for cohort studies [57]. Consistently, Mariani et al., found n-3 PUFAs consumption to be not significantly associated with incident AF in 9354 subjects from both RCT and observational studies, with a pooled risk ratio of 0.9 (95%CI 0.79–1.13) for AF recurrence and 0.86 (95%CI 0.71–1.04) for postoperative AF [58]. In seven prospective cohort studies covering 206,811 participants, Li et al., confirmed that the highest versus lowest category of fish consumption and dietary intake of n-3 PUFAs was not significantly associated with lower risk of AF [59].

It should be acknowledged that variations in the dose of n-3 PUFAs among the groups across the studies and different methods for assessing n-3 intake were important limitations of the above-mentioned works and that the amount and type of fish/PUFAs intake are factors that must be considered in future studies.

The Diet, Cancer, and Health Cohort Study that included a population of 57,073 patients prospectively followed for a mean of 13.6 years, showed that a higher consumption of marine n-3 PUFAs was associated with a lower AF risk with a U-shaped association describing greater benefits from a moderate intake of 0.63 g/day [60]. In another study on 5000 adults, the consumption of tuna or other broiled or baked fish more than five times per week had an inverse association with the incidence of AF, with a 31% risk reduction, which was not observed with fried fish consumption [61].

The effect of extra virgin olive oil (EVOO) on AF occurrence/recurrence has been investigated in 6705 patients from the Prevention With Mediterranean Diet (PREDIMED) trial that showed a protective effect of a Med-Diet enriched with EVOO on new-onset AF in primary prevention; thus, EVOO significantly reduced the risk of newly detected AF in a median follow-up of 4.7 years (hazard ratio [HR], 0.62; 95%CI 0.45–0.86) [62].

Two studies investigated nut consumption and incident risk of AF. Larsson et al., reported a linear and dose–response inverse association of nuts consumption with AF (*p* for trend = 0.004) even after adjustment for multiple risk factors, with a risk reduction of 18% for nuts consumption ≥ 3 times/week [63]. Conversely, nut assumption was not associated with a risk of AF in the Physicians’ Health Study, which was a prospective cohort of 21,054 male physicians in which no statistically significant association between nut consumption and AF was found when stratified by body mass index or age [64].

In the past few years, several prospective studies have investigated the protective role of coffee and chocolate intake for AF occurrence (Table 2). The most important finding is probably that their effect is modulated by the daily amount of their intake. Three prospective studies emphasized the beneficial effect of moderate coffee consumption of 1–2 cups per day [65,66,67]. Conen et al., prospectively followed 33,638 women aged >45 years for 14 years and found a 22% risk reduction for subjects with 285 mg of caffeine consumption per day (about two cups of coffee), as compared with subjects with no/low intake [65]. Similarly, Bodar et al., reported a 14% of risk reduction for a 2–3 coffee cups/day consumption in a prospective study of 18,960 adults [66]. More recently, Bazal et al., meta-analyzed results from SUN (18,983 adults) and PREDIMED (6479) studies, finding a 40% risk reduction for intermediate coffee consumption of 1–7 cups/week, while higher levels of caffeinated coffee consumption (>1 cup per day) had no significant effect [67]. On the other hand, only one study, enrolling 57,053 adults aged 50–64 years old, showed a high consumption of 6–7 cups per day of coffee was significantly associated with a lower risk of developing AF (HR 0.79, 95%CI 0.64–0.98) [68].

As for chocolate, two out of three prospective studies indicated no influence of chocolate intake on AF risk [69,70,71]. The largest study that involved >50,000 adults with 13.5 years of follow-up showed a favourable antiarrhythmic effect of 2–6 servings/week (30 g of chocolate per serving; HR = 0.80, 95%CI 0.71–0.91) [70].

In Table 2, we also report foods that have been reported to increase the risk of incident AF. The role of alcohol consumption in AF risk was investigated in a recent dose–response meta-analysis, confirming the detrimental effect of moderate–high alcohol consuming with an incremental RR of 1.47 (95%CI 1.34–1.61) for five drinks/day [72]. As a matter of fact, experimental studies showed electrical and structural changes in regular drinkers that may explain the propensity to AF of these subjects associated with conduction slowing and a lower atrial voltage [73]. Lastly, fried foods, salt intake and low-carbohydrate diets seem also to enhance AF risk but poor data are available to date (Table 2) [74,75,76]. For example, there are many processes in which low carbohydrate diets might lead to the onset of AF. It is suggested that people following low carb diets might also consume fewer grains, fruits, and vegetables which have well-known antioxidant properties. Few studies have examined the relationship of carbohydrate intake and risk of incident AF, finding that a low-carbohydrate intake is associated with an increased risk of incident AF, regardless of the type of protein and fat used to replace the carbohydrate [76].

**Table 2:** Prospective studies and randomize controlled trials exploring the association between single foods/nutrients and incident atrial fibrillation.

Food/Nutrient	Author, Year	Population (n)	Study Design	Follow-Up (Years)	Main Findings
Fish and omega-3 PUFA					
Tuna or broiled/baked fish	Mozaffarian et al., 2004 [61]	4815 adults ≥ 65 years old	P	12	Consumption of tuna or other broiled or baked fish was inversely associated with incidence of AF: with an intake of 1 to 4 times per week (HR 0.72, 95%CI 0.58–0.91; *p* = 0.005) and intake 5 times per week (HR 0.69, 95%CI 0.52–0.91, *p* = 0.008), compared with 1 time per month (*p* trend 0.004). Fried fish/fish sandwich consumption was not associated with AF.
Omega-3 PUFAs	Brouwer et al., 2006 [77]	5184 adults	P	6.4	Intake of EPA and DHA in the third tertile compared with first was not associated with risk of AF (RR 1.18, 95%CI 0.88–1.57). No association was observed with intake of >20 g/day fish compared with no fish intake (RR 1.17, 95%CI 0.87–1.57).
Omega-3 PUFAs	Shen et al., 2011 [78]	4526 adults	P	4	No significant association between n-3 (omega-3) PUFAs and AF risk: Q4 1.11 (95%CI 0.81, 1.54); Q3 0.92 (95%CI 0.65, 1.29); Q2 1.18 (95%CI 0.85, 1.64); *p* for trend 0.57, Q1 as reference group.
Fish and omega-3 PUFAs	Rix et al., 2014 [60]	57,053 adults aged 50–64 years old	P	13.6	Q1 as reference group: Q2 HR 0.92 (95%CI 0.82–1.03); Q3 HR 0.87 (95%CI 0.78–0.98); Q4 HR 0.96 (95%CI 0.86–1.08); Q5 HR 1.05 (95%CI 0.93–1.18) Intake of total fish, fatty fish, and the individual n-3 PUFA EPA, DHA, DPA also showed U-shaped associations with incident AF.
Fish and omega-3 PUFAs	Larsson and Wolk. 2017 [79]	72,984 adults aged 45–83 years old	P	12	Intake of total fish, fatty fish (herring/mackerel and salmon/whitefish/char), and omega-3 PUFAs not associated with AF incidence after adjustment for risk factors. High consumption of lean fish (cod/saithe/fish fingers) associated with a lower risk: HR 0.79, 95%CI 0.65–0.95).
Extra virgin olive oil					
Extra virgin Olive Oil	Martínez-González, 2014 [62]	6705 adults	RCT	4.7	Participants assigned to Mediterranean diet supplemented with extravirgin olive oil had a lower risk of AF development (HR 0.62; 95%CI 0.45–0.8) after adjusting for propensity scores.
Nuts					
Nuts	Khawaja et al., 2012 [64]	21,054 males	P	20	Multivariable adjusted HR for incident AF were 1.00 (95%CI 0.90–1.11), 1.09 (95%CI 0.97–1.21), 1.07 (95%CI 0.95–1.21), and 0.91 (95%CI 0.70–1.17) for nut consumption from the lowest to the highest category of nut consumption (*p* for trend 0.26).
Nuts	Larsson et al., 2018 [80]	61,364 adults	P	17	Nut consumption ≥ 3 times/week inversely associated with AF in the age-adjusted and sex-adjusted analysis (HR 0.87, 95%CI 0.67–0.89, *p* linear trend 0.002). Compared with no consumption of nuts, the multivariable HRs of AF across categories of nut consumption were not significant different 0.97 (95%CI 0.93–1.02) for 1–3 times/month, 0.88 (95%CI 0.79–0.99) for 1–2 times/week and 0.82 (95%CI 0.68–0.99) for ≥ 3 times/week.
Coffee					
Coffee	Conen et al., 2010 [65]	33,638 women > 45 years old	P	14.4	Median caffeine intake across increasing quintiles of caffeine intake were 22, 135, 285, 402, and 656 mg/d, respectively. In Cox proportional hazards models, the adjusted HR were 0.88 (95%CI 0.72–1.06) for Q2, 0.78 (95%CI 0.64–0.95) for Q3, 0.96 (0.79–1.16) for Q4, and 0.89 (0.73–1.09) for Q5 (*p* for linear trend 0.45). None of the individual components of caffeine intake (coffee, tea, cola, and chocolate) were significantly associated with incident AF.
Coffee	Mostofsky et al., 2016 [68]	57,053 adults 50–64 years old	P	13.5	Coffee consumption inversely associated with AF incidence with multivariable-adjusted HR of 0.93 (95%CI 0.74–1.15) for more than none to <1 cup/day, 0.88 (95%CI 0.71–1.10) for 1 cup/day, 0.86 (95%CI 0.71–1.04) for 2–3 cups/day, 0.84 (95%CI 0.69–1.02) for 4–5 cups/day, 0.79 (95%CI 0.64–0.98) for 6–7 cups/day and 0.79 (95%CI 0.63–1.00) for >7 cups/day (*p*-linear trend 0.02).
Coffee	Bodar et al., 2019 [66]	18,960 adults	P	9	HR (95%CI) of AF were 0.85 (95%CI 0.71–1.02) for ≤1 cup/week, 1.07 (95%CI 0.88–1.30) for 2–4 cups/week, 0.93 (95%CI 0.74–1.17) for 5–6 cups/week, 0.85 (95%CI 0.74–0.98) for 1 cup/day, 0.86 (95%CI 0.76–0.97) 2–3 cups/day, and 0.96 (95%CI 0.80–1.14) for 4+ cups/day, reference group was coffee consumption of rarely/never (*p* for nonlinear trend 0.01). In a secondary analysis the multivariable adjusted HR of AF per standard deviation (149 mg) change in caffeine intake was 0.97 (95%CI 0.92–1.02).
Coffee	Bazal et al., 2021 [67]	18,983 adults from SUN and 6479 from PREDIMED cohorts	P	10.3 SUN and 4.4 PREDIMED	An intermediate level of coffee consumption (1–7 cups/week) was inversely associated with the risk of AF in the PREDIMED study, compared with participants drinking < 3 cups/month (47% RR reduction, 95%CI 21–64%). In the SUN cohort no statistically significant association was found. The meta-analysis of both studies showed a 40% RR reduction (95%CI 18–56%) of coffee consumption and AF risk compared with participants drinking < 3 cups/month. In the meta-analysis of both PREDIMED and SUN studies, the HR for intermediate consumption of coffee was 0.60 (95%CI 0.44–0.82).
Chocolate					
Chocolate	Khawaja et al., 2015 [69]	18,819 male physicians	P	9	Using <1/month of chocolate consumption as the reference group, multivariable adjusted HR for AF were 1.04 (0.93–1.18) for chocolate intake of 1–3/month, 1.10 (0.96–1.25) for 1/week, 1.14 (0.99–1.31) for 2–4/week, and 1.05 (0.89–1.25) for ≥5/week (*p* for trend 0.25), with no intake as reference group.
Chocolate	Mostofsky et al., 2018 [70]	55,502 adults	P	13.5	Rate of AF was lower for people consuming 1–3 servings/month (HR = 0.90, 95%CI 0.82–0.98), 1 serving/week (HR = 0.83, 95%CI 0.74–0.92), 2–6 servings/week (HR = 0.80, 95%CI 0.71–0.91) and 1 servings/day (HR = 0.84, 95%CI 0.65–1.09; *p* linear trend <0.0001), with chocolate intake less than once per month as reference group.
Chocolate	Larsson et al., 2017 [71]	9978 adults	P	14.6	Compared with non-consumers, the multivariable HR of AF for those in the highest category of chocolate consumption (≥3–4 servings/week) was 0.96 (95%CI 0.88–1.04).
Alcohol					
Alcohol	Frost and Vestergaard 2004 [81]	47,949 adults	P	5.7	Adjusted HR in men were 1.04, 1.44, 1.25, and 1.46 for quintiles Q2, Q3, Q4, and Q5 (*p* for trend 0.04), with Q1 as reference group. In women, there did not seem to be any association between consumption of alcohol and risk of AF.
Alcohol	Conen et al., 2008 [82]	34,715 women	RCT	12.4	Compared with nondrinking women, women consuming 2 or more drinks per day had an absolute risk increase of 0.66 events/1000 person years. The multivariate-adjusted HRs for incident AF were 1.05 (95%CI 0.88–1.25) for more than 0 and less than 1 per day, 0.84 (95%CI 0.58–1.22) for 1 or more and less than 2, and 1.60 (95%CI 1.13–2.25) 2 or more drinks per day. The increased hazard in the small group of women consuming 2 or more drinks per day persisted when alcohol intake was updated at 48 months (HR 1.49; 95%CI 1.05–2.11) or when women were censored at their first cardiovascular event (HR 1.68; 95%CI 1.18–2.39).
Alcohol	Liang et al., 2018 [83]	30,433 adults 55 years or older	RCT	4.5	Compared with participants who had a low level of consumption, those with higher levels had an increased risk of incident AF (adjusted HR 1.14, 95%CI 1.04–1.26, for moderate consumption; 1.32, 95%CI 0.97–1.80, for high consumption). Results were similar after we excluded binge drinkers. Among those with moderate alcohol consumption, binge drinkers had an increased risk of atrial fibrillation compared with non-binge drinkers (adjusted HR 1.29, 95%CI 1.02–1.62).
Alcohol	Larsson et al., 2014 [72]	79,019 adults	P	12	The association between alcohol consumption and AF did not differ by sex (*p* for interaction 0.74). Compared with current drinkers of <1 drink/week (12 g alcohol/drink), the multivariable RRs of AF were 1.01 (95%CI 0.94–1.09) for 1 to 6 drinks/week, 1.07 (95%CI 0.98–1.17) for 7 to 14 drinks/week, 1.14 (95%CI 1.01–1.28) for 15 to 21 drinks/week, and 1.39 (95%CI 1.22–1.58) for >21 drinks/week. Results were similar after excluding binge drinkers. In a meta-analysis of 7 prospective studies the RRs were 1.08 (95%CI 1.06–1.10) for 1 drink/day, 1.17 (95%CI 1.13–1.21) for 2 drinks/day, 1.26 (95%CI 1.19–1.33) for 3 drinks/day, 1.36 (95%CI 1.27–1.46) for 4 drinks/day, and 1.47 (95%CI 1.34–1.61) for 5 drinks/day, compared with non-drinkers.
Carbohydrate					
Carbohydrate	Zhang et al., 2019 [76]	13,385 adults	P	22.4	The HR for incident AF associated with a 1-SD (9.4%) increase in carbohydrate intake as a percentage of energy intake was 0.82 (95%CI 0.72–0.94), after adjustment for traditional AF risk factors and other diets factors. In the final model, the HR for incident AF comparing the second, third, and fourth quartiles of carbohydrate intake as a percentage of energy with the first quartile were 0.79 (95%CI 0.68–0.92), 0.77 (95%CI 0.64–0.93), and 0.64 (95%CI 0.49–0.84) separately.
Fried foods and salt intake					
Fried foods	Khawaja et al., 2020 [74]	18,941 males	P	9.0	Multivariable adjusted HR for AF were 1.07 (95%CI 0.97–1.18) for fried food consumption of 1–3/week and 1.03 (95%CI 0.91–1.17) for ≥4/week (*p* linear trend 0.4) (<1/week as reference group).
Salt	Wuopio et al., 2021 [75]	473,080 adults	P	10	Adjusted model showed significant associations amongst men in the lowest and highest quintiles of sodium excretion (HR Q1 1.20, 95%CI 1.08–1.32, *p* < 0.001 and HR Q5 1.15, 95%CI 1.03–1.27, *p* = 0.011).

AF: atrial fibrillation; CI: confidence interval; DHA: docosahexaenoicacid; DPA: docosapentaenoic acid; EPA: eicosapentaenoic acid; HR: hazard ratio; P: prospective; PREDIMED: Prevencion con Dieta Mediterranea; PUFA: poly-unsaturated fatty acids; RCT: randomized controlled trial; RR: relative risk; SD: standard deviation; SUN: Seguimiento Universidad de Navarra.

## 5. Effects of Med-Diet on MetS Components and NAFLD

The Med-Diet is a cornerstone for the treatment of cardiovascular risk factors that define the MetS. The PREvención con DIeta MEDiterránea (PREDIMED) study, performed with 7447 men and women at high risk for cardiovascular disease followed for 4.8 years, showed around a 30% reduction in cardiovascular events in patients treated with a standardized Med-diet [84]. In addition, this study showed a significant improvement in cardiovascular risk factors such as high blood pressure, insulin resistance, abnormal lipid profiles, all driven by Med-Diet [84].

The Med-Diet, similarly to the Dietary Approaches to Stop Hypertension (DASH) diet, ketogenic diet and intermittent fasting diet, induces weight loss [85].

Med-Diet is characterized by a high intake of unsaturated fat-rich oils (sunflower, rapeseed, corn, olives). These were shown to reduce triglycerides when used in the substitution of saturated fatty acids-rich foods such as butter or lard [86] improving lipid profile. Triglycerides serum levels may be also improved by a high fibre intake contained in the Med-Diet, especially in patients with diabetes mellitus type 2 and MetS [87,88].

In addition, the Med-diet could improve high arterial blood pressure [89]. This is supported by a randomized clinical trial [90] and a large systematic review and meta-analysis [91] that show a reduction of 24 h ambulatory blood pressure in patients taking Med-Diet independently from sodium intake.

Finally, the Med-Diet may improve diabetes mellitus [90], indeed, it is characterized by food with a poor glycaemic index and can improve insulin resistance and weight loss resulting in a better control of glycaemic profile [92].

The Med-Diet may also have a beneficial effect on NAFLD. As far as there is no pharmacological treatment approved for the treatment of NAFLD, diet modifications represent the best approach.

General recommendations include energy restriction, abstention from fructose and alcohol intake, physical activity improvement and a 7–10% weight loss [93].

Although there are some diets useful to achieve these therapeutic goals (DASH diet, ketogenic diet, intermittent fasting diet) [85], Med-Diet plays a role in prevention of hepatic steatosis [94]. For instance, the high content of PUFAs could reduce insulin resistance and intrahepatic triglyceride content, thereby improving NAFLD [92].

A RCT that included 50 overweight NAFLD patients randomized to receive a Med-Diet with or without antioxidant supplementation, showed an improvement in anthropometric parameters, lipid profile and reduced hepatic fat accumulation and liver stiffness [95].

An RCT including adolescents with NAFLD randomized to receive Med-Diet showed a significant improvement of liver function tests, hepatic steatosis, insulin resistance and oxidative stress after 12 weeks [96].

As mentioned before, the antioxidant effect of Med-Diet may be particularly useful for NAFLD patients, as oxidative stress plays a pivotal role in the onset and progression of NAFLD. Indeed, in patients with NAFLD, a poor adherence to a Med-Diet has been associated with high serum levels of sNox2-dp and serum lipopolysaccharide (LPS) [97].

This last finding is of particular interest, as gut-derived LPS has been shown to localize into fatty liver and contribute to hepatic inflammation [98]. Moreover, LPS promotes insulin resistance, a key pathogenetic mechanism for NAFLD onset. This is of interest considering that LPS has been associated with an increased risk of CVEs in different clinical scenarios, including AF [99].

## 6. Med-Diet, Oxidative Stress and Cardiovascular Events in AF

Even if there are no available data from RCTs of diet administration specifically performed in patients with AF with or without MetS or NAFLD, there are few data coming from observational studies regarding the association between different degrees of adherence to Med-Diet and incidence of CVEs in AF.

One of the past concerns about the recommendation of Med-Diet as an antioxidant therapeutic approach in AF patients was due to the presence, in this diet regimen, of a large amount of vegetables rich in vitamin K that could interfere with the anticoagulant activity of vitamin K antagonist. The first finding that should be underlined is that despite evidence that vitamin K supplementation may interfere with anticoagulation (for an intake of vitamin K > 150 µg/day) [100], adherence to a Med-Diet does not seem to have any clinically relevant effect on anticoagulation stability [101].

One easy tool to assess the adherence to the Med-Diet is a well validated questionnaire that takes into account the weekly use of cardioprotective foods/nutrients such as EVOO, fruit, vegetables, legumes, fish, and red wine and potentially pro-inflammatory aliments such as red meat, rice and bread [102]. The score obtained allows for the classification of patients in high (7–9 points), moderate (4–6 points) and low adherence (0–3 points) groups. In a study on 709 AF patients, after a median follow up of 40 months, 72 MACE occurred: 23.4% in low, 8.4% in intermediate and 5.3% in high adherence group [103]. The survival analysis showed a significant decrease in the rate of CVEs across the three Med-Diet’s adherence groups (Log-Rank Test, *p* < 0.001) [103].

In support of the protective role of Med-Diet, another study of 900 non valvular AF patients found that Med-Diet when associated with regular physical activity was associated with a lower risk for silent cerebral ischemia detected by magnetic resonance in the group of AF patients with low Med-Diet adherence and no physical activity [104].

Improvement of oxidative status has been hypothesized as a potential mechanism accounting for the cardiovascular protection by Med-Diet.

The relationship between Med-Diet, oxidative status and CVEs in AF patients has been investigated in the prospective Atherosclerosis in Atrial Fibrillation (ATHERO-AF) cohort. In particular, the study showed that Med-Diet score inversely correlated with serum Nox2 (Rs: −0.297, *p* < 0.001), F2-Isoprostanes (Rs: −0.411, *p* < 0.001) [103]. The analysis of antioxidant enzymes activity showed that GPx3 was directly associated with Med-Diet score with a lower effect on SOD levels [105]. The reduction of CVEs coincidentally with an improvement in the oxidative status leads to the hypothesis that a high adherence to the Med-Diet could be associated with a reduction in CVEs.

## 7. Conclusions and Open Issues

The mechanisms through which a Med-Diet may reduce cardiovascular risk in patients with AF and MetS or NAFLD are multiple, including via the prevention of cardiovascular risk factors, lowering oxidative stress and insulin resistance and improving antioxidant status.

The reduction in CVEs associated with a high adherence to Med-Diet, coincidentally with an improvement in oxidative stress suggests that this may represent an important mediator in cardiovascular risk. However, all available evidence comes from observational studies and needs to be confirmed by ad hoc RCT.

Despite this favourable evidence on the role of Med-Diet, there are still several open research fields. For instance, it remains to be well established whether an improvement in systemic oxidative stress in AF patients may result in lower NAFLD or progression to NASH and conversely, if a modulation of insulin resistance by Med-Diet in MetS/NAFLD patients may have a favourable impact on reducing new-onset AF.

## Data Availability

Not applicable given the study type.
